# Types of refractive errors in a sample of Iraqi children with Intermittent exotropia

**DOI:** 10.12688/f1000research.156932.2

**Published:** 2024-12-09

**Authors:** Najah K. Mohammad, Ibrahim Ali Rajab

**Affiliations:** 1 Department of Surgery, College of Medicine, University of Baghdad, Baghdad, Iraq

**Keywords:** Intermittent exotropia, emmetropia, myopia, refractive errors, pediatric strabismus.

## Abstract

**Background:**

One of the most common strabismus types in children is intermittent exotropia, which predominantly occurs in children between the ages of 2 to 4 years. It may affect visual development and often coexists with refractive errors. Unlike esotropia, which usually links to hyperopia, intermittent exotropia might have a different relationship with refractive errors and thus lead to myopia.

**Methods:**

In this cross-sectional retrospective study from August 2021 to December 2023, 179 patients diagnosed with intermittent exotropia were recruited via an outpatient clinic of Najah Al-Quraishi, Baghdad, Iraq. The refractive errors were compared by autorefractometry and retinoscopy after cycloplegic dilation. Data analysis was constructed under the use of IBM SPSS V.26 for the determination of emmetropia, myopia, and hyperopia prevalence.

**Results:**

Among the 179 patients, emmetropia was the most commonly observed refractive status, present in 68 patients (38%). Low hyperopia and low myopia were also common, with 64 and 40, respectively. A limited number of patients had moderate/high myopia and moderate/high hyperopia; in detail, the prevalence was following: 5% of patients had moderate myopia, while 0.5% of patients suffered from high myopia; symmetrically, 5% were moderate to high hyperopia. From the data collected, a trend emerged for a low refractive error and symmetric refractive error in both eyes.

**Conclusion:**

Contrasted to prior conventional wisdom about refractive error in strabismus, it established the greater incidence of emmetropia in patients with intermittent exotropia. The findings call for specific management strategies to be applied in this population.

## Introduction

Intermittent exotropia is an active type of strabismus that affects quite a large number of children. However, its prevalence might vary from one ethnic and age group to another. ^1, 2^ Intermittent exotropia is characterized by periodic outward deviation of one eye, which may disturb binocular vision in some patients. This has immense implications for visual development and the quality of life. The complicated relationship between intermittent exotropia and refractive errors has been known, though not so much understood; according to some studies, different patterns of refractive anomalies could exist in these patients. ^1, 3^

Strabismus prevails in approximately 5%-8% of the general population; among these, exotropia ranks as one of the most common varieties, mainly between ages 2-4 years. ^1, 3^ Intermittent exotropia is probably considered the most tricky subset of exotropia in terms of coexistent refractive error and management. Whereas esotropia can be associated with plus, the relationship between exotropia and refractive errors is more complex. Of importance, many children with intermittent exotropia become myopic over time; this has been reported as greater than 90% in some cohorts by some series. ^4^

In order to better understand the therapy and prognosis of intermittent exotropia, this study will look into the distribution of refractive errors in children and young people with the illness. It will also look for common refractive patterns.

## Methods

### Study design and setting

This is a cross-sectional retrospective study that took place in the corresponding author’s private ophthalmology clinic. The clinic serves a diverse population, primarily children from various walks of life suffering from ocular conditions including intermittent exotropia. This being an observational study, there is no control, hence all eligible patients diagnosed with intermittent exotropia within the study period ranging from August 2021 to December 2023 were included.

Patients were enrolled consecutively from the routine clinic visits. All subjects had been referred to the study from the clinics for clinical presentations of intermittent exotropia and, therefore, represented a homogenous population by diagnosis. No external advertisements for patient recruitment were utilized, and consecutive eligible patients presenting at the clinic were included, with further selection based only on exclusion criteria regarding previous ocular surgery or other types of strabismus.

Refractive errors were determined by autorefractometry, using a Topcon KR-800 ^®^^,^™ (Topcon Corporation, Tokyo, Japan) following cycloplegia with two drops of 1% cyclopentolate given 10 minutes apart. Testing was done 30 minutes after the final drop to ensure that complete cycloplegia had been achieved. All patients underwent three successive refractometry examinations on each eye to reduce variability. The amount of exotropia was measured by prism cover test. All equipment was calibrated according to the manufacturer’s guidelines before use and checked regularly during the study for accuracy.

This study followed the STROBE checklist for cross-sectional studies. ^5^ The study design, setting, participants, variables, data sources, measurement techniques, and statistical methods are those in the list that apply and have been appropriately reported to ensure the reproducibility and transparency of the study. Therefore, a completed STROBE checklist is attached and provided to ensure verification of standards.

### Participants

This study only included patients whose inclusion criteria consisted of a proven clinical diagnosis of intermittent exotropia, ages from childhood to early adulthood, and those who gave their assent to participate in this research study. The exclusion criteria for this study include those patients with a history of ophthalmic surgery, all constant forms of strabismus, secondary exotropia ensuing from other eye pathology, and systemic conditions that affect visual function.

Squint division into two age groups, namely childhood below six years and school age six years or above, goes in line with the consideration of refractive errors. Both problems are of great educational and psychosocial concern to these age groups, especially since the starting age for school is six years old in Iraq. ^6, 7^

### Sample size determination

The total number of participants involved in this study was calculated according to the number of patients who presented with intermittent exotropia in the consultation at the outpatient clinic from August 2021 to December 2023. It is an exhaustive sample of all eligible patients who visited with a diagnosis of intermittent exotropia from August 2021 to December 2023. 179 patients were included to be eligible out of 195 and the rest were excluded mainly for non-sufficient data.

### Data collection

Refractive errors were then categorized as emmetropia (±0.50 diopters), myopia (< -0.50 diopters), and hyperopia (> +0.50 diopters). In addition, low myopia/hyperopia was considered to be up to 3 diopters of error, while moderate/high was beyond this range. Refractive errors were diagnosed by routine ophthalmic tests, including auto-refractometry and retinoscopy after cycloplegia induced by cyclopentolate. The prism cover test found the amount of exotropia. The data collected were then classified and tabulated according to the refractive status of both eyes – emmetropia and myopia are classified into low (less than three diopters), moderate (3 – 6 diopters), and high (more than six diopters). Still, the divisions of hypermetropia are not distinct in the readings accept that follows the similar rating scale depending on the magnitude of refractive error. ^8^ The Ethics Committee (Iraqi Board of Medical Specializations) further stated that the current research study does not require approval because it is a retrospective and has no intervention. Moreover, verbal consent was obtained from all participants in this study instead of written consent. This approach was chosen for several reasons. First, many participants had low literacy levels, making written consent impractical and potentially exclusionary. Second, cultural sensitivities within the community associated signing documents with legal issues or distrust, so verbal agreements were more culturally appropriate and helped build rapport. Written consent was waived since the data in this study were retrospectively analyzed from medical records, and no direct contact with the patients was required to collect any data. However, in regard to further data collection from the patients during follow-up visits and that of minors, verbal consent was obtained, approved by the Ethics Committee. In the participants below 18 years of age, consent was obtained from their parents or guardians. Verbal consent was documented due to cultural and logistical barriers that made obtaining written consent impractical for some participants.

### Statistical analysis

Data analysis was done using IBM SPSS V.26, where descriptive statistics summarized data and tabulated results in accounts and percentages of each category of refractive error. Another level summary of analysis was undertaken by calculating the mean for both the spherical equivalence and the cylindrical and spherical correction outcome of either eye. These were also tabulated and interpreted to make sense of the refractive trends in this population of patients.

The data were checked for completeness and accuracy before statistical analysis; partial refraction records and those values that did not logically fit the established norms, after cross-checking with the patients’ records, were screened out. The outliers were defined as those values that lay beyond three standard deviations from the mean of the distribution and were reviewed for possible recording errors. These were included if clinically relevant, as this reflected extreme cases of refractive errors. Sensitivity analyses excluding these outliers were conducted to see the robustness of the results. Overall analysis was conducted using IBM SPSS V.26, with descriptive statistics conducted to summarize patient demographics and refractive error distributions.

Normality was checked by the Shapiro-Wilk test to decide which set of statistics tests should be applied. Parametric tests were conducted for normally distributed data; otherwise, non-parametric tests were conducted. Outliers, defined as values more than three standard deviations away from the mean, were removed from analysis after confirmation of their non-representative nature. Descriptive statistics summarized data by using means and standard deviations for continuous variables and percentages for categorical variables. All statistical analyses were performed using IBM SPSS V.26.

## Results

A total of 179 patients with intermittent exotropia were included in this study. Of these, 96 (47.5%) were male, while 67 (37.4%) were children, and 112 (62.6%) were young students. The demographics of the study participants are summarized in Table 1.

**Table 1.** Demographics of study participants.

Demographic variable	Category	Count	Percentage (%)
Gender	Male	96	47.5
	Female	83	52.5
Age group	Children (below 6)	67	37.4
	Young students (6+)	112	62.6
Total		179	100

### Prevalence of refractive errors

**Figure 1.**
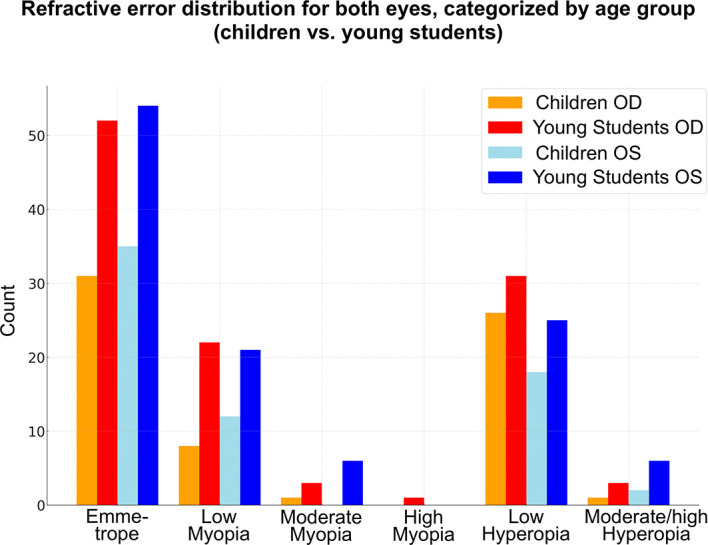
Heatmap of refractive errors between the two eyes.

Emmetropia was the most common refractive error, observed in 68 eyes (38%). Low hyperopia and low myopia followed, affecting 64 (35.7%) and 40 (22.3%) patients, respectively. Moderate myopia was present in 9 patients (5%), high myopia in 1 patient (0.5%), and moderate to high hyperopia in 9 patients (5%) ( Figure 1).

### Symmetry in refractive errors

**Figure 2.**
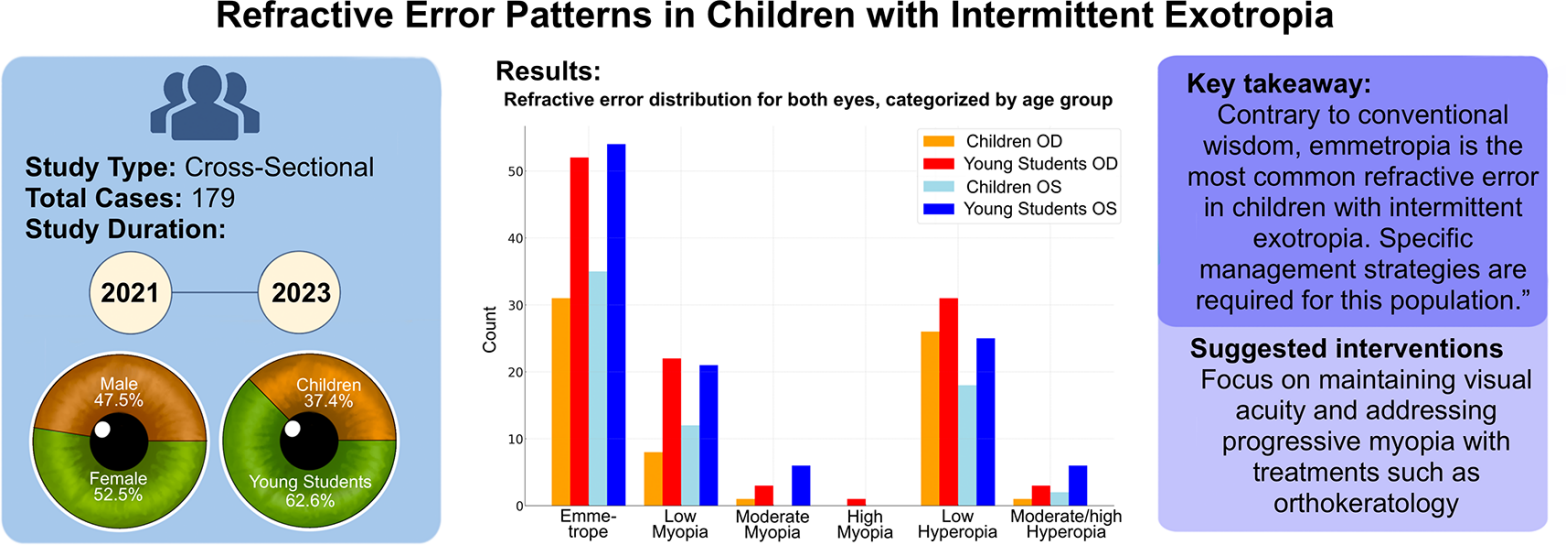
Graphical design summarizing the study results.

A cross-tabulation of the refractive errors between the two eyes showed a high degree of symmetry ( Figure 2). Most patients had very similar refractive errors in both eyes. Emmetropic and low myopic patients showed a robust bilateral correlation in refractive errors. All moderate to high refractive errors, although less symmetrical, were still recorded in some minority of patients ( Table 2).

**Table 2.** Cross-tabulation of refractive errors between the two eyes.

		Emmetrope	Low myopia	Moderate myopia	High myopia	low hyperopia	moderate/high hyperopia
		Count	Count	Count	Count	Count	Count
Refractive error OS	Emmetrope	68	5	2	0	14	0
	Low myopia	7	23	1	0	2	0
	Moderate myopia	2	2	1	1	0	0
	High myopia	0	0	0	0	0	0
	Low hyperopia	6	0	0	0	36	1
	Moderate/high hyperopia	0	0	0	0	5	3

### Detailed refractive error analysis

Emmetropia showed a mean spherical equivalence of 0 for both eyes. Low myopia had a mean spherical equivalence of -2 for both eyes, while moderate myopia showed a mean spherical equivalence of -4 for OD and -4 for OS. High myopia was rare, with only one case, showing a mean spherical equivalence of -6 for OD and not applicable for OS. Low hyperopia had a mean spherical equivalence of 2 for both eyes, and moderate/high hyperopia had a mean spherical equivalence of 5 for OD and 4 for OS.

## Discussion

Probably the most striking fact brought out by this study of intermittent exotropia—an eye misalignment characterized by outward deviation—is the remarkably high prevalence of emmetropia, wherein fully 50% of the patient sample showed no refractive error. ^9^ This rate not only forms the bulk of the refractive statuses but contrasts significantly with the expected rates for myopia and hypermetropia traditionally associated with strabismic conditions. This high quantity of emmetropia strongly suggests that intermittent exotropia does not follow the usual patterns of refractive error seen in most other strabismus.

This study ranges from 20 to 23% in terms of the prevalence of myopia among the cohort, standing in striking contrast to the 55.5% of myopia prevalence in the cohort studied by Robaei et al. ^10^ This discrepancy may reflect demographic, genetic, or environmental differences between the populations or, perhaps more likely, differing criteria for classifying myopia. Our rates are more comparable to those found in a Pakistani study, which also reported an exotropic population with a 50% rate of emmetropia and 29.4 % myopia. ^11^

Our research further elaborates on the relationship between hyperopia and intermittent exotropia. While this association has been reported constantly, our findings give further sophistication to this understanding in pointing out that hyperopia is not as frequent as emmetropia or myopia within this type of strabismic condition. This makes us think about thought whether the refractive error profile for intermittent exotropia might differ from other strabismic disorders in which hyperopia shows up more frequently. ^12– 14^

The paper also discusses the refractive error as dynamic. In particular, it focuses on the trend of an increase in myopia among children who are diagnosed with intermittent exotropia. ^15^ This can indicate that developing myopia is not the cause for exotropia. Still, rather the condition of intermittent exotropia itself could be one of the causes that contribute to the development of myopia. It has been hypothesized that this increased accommodative demand that accompanies the effort at maintaining binocular alignment in intermittent exotropia could very well explain its progression into myopia. ^16^ This is corroborated by studies showing that treatments meant to lessen accommodation, like wearing bifocal glasses, impede the advancement of intermediate myopia. ^17^

Given our findings, therapeutic strategies for addressing refractive errors in intermittent exotropia patients have to be tailored accordingly. Since the most significant proportion accounted for was emmetropia and myopia and hyperopia relatively less common, treatments must be directed toward maintaining good visual acuity and possible changes? For those who have myopia, particularly progressive forms, interventions such as orthokeratology may be well worth considering. What should always be put forward in any management for intermittent exotropia is the fact that comprehensive care embodies both correction for strabismus and management for refractive error. ^18^

These findings are in agreement with reports of previous studies that intermittent exotropia may have a refractive error profile different from other forms of strabismus. For example, the relatively high prevalence of emmetropia and low hyperopia in our cohort, compared to the types usually observed among esotropic patients, underlines this distinct distribution of refractive errors and further justifies the need for diagnostic and therapeutic strategies specific to intermittent exotropia. ^19, 20^ This differentiation of treatment strategies is essential for the designation of intermittent exotropia as an independent clinical entity, outlining its characteristics of refraction. ^21^

Our study also opens the window for further understanding of how these refractive error patterns may influence long-term visual outcomes. The relative incidence of high myopia and hyperopia may be low, and the prognosis for intermittent exotropia might turn out to be relatively favorable when compared to those with constant esotropia or exotropia if timely and proper measures are implemented. ^22^ This fact heightens concern for regular ophthalmologic follow-up examination and early corrective measures to avoid the progression of refractive errors and achieve optimum visual development. ^23^ Longitudinal studies in the future must be directed toward understanding the progression of refractive error in intermittent exotropia and setting up evidence-based guidelines for its management. ^24, 25^

This current study, hence, has two significant implications: an alternative view of the distribution of refractive error in intermittent exotropia and a causative relationship between strabismus and myopia development. Herein lies critical insight as to which targeted treatment methods may be tailored to treat not only strabismus itself but also consider the potential of growing refractive errors. The high prevalence of emmetropia in intermittent exotropia, as seen in this study, needs to be put into the proper perspective of refractive error distribution in populations with and without strabismus. Such a comparison is important to establish whether this prevalence represents a particular characteristic of IXT or part of a general trend seen in normal binocular vision.

However, the limitations of our study merit mention. One major limitation in the drawing of solid conclusions regarding the causality of intermittent exotropia and refractive errors is not having a control group of nootropic subjects. Second, there is still missing longitudinal data defining the temporal development of refractive errors relative to the onset and progression of intermittent exotropia. Although our retrospective design did not allow for a normal binocular vision control group, such a comparison would indeed be very valuable in the future, especially in the prospective studies that would like to tease out whether this prevalence is a unique feature of intermittent exotropia or rather part of the broader pattern of refractive error. This paper thus sheds light on the refractive landscape of intermittent exotropia. Still, it makes an even stronger case for other and more comprehensive studies to tease out complicated interactions between refractive errors and this common form of strabismus.

## Conclusions

Our study showed that contrary to the traditionally expected predominance of myopia and hyperopia in strabismus, the most frequent refractive errors in intermittent exotropia patients are emmetropia, followed by hypermetropia. Therefore, this might suggest that rather than the reverse, intermittent exotropia is perhaps one of the underlying predisposing factors for myopia development.

## Ethics and consent

The Ethics Committee (Iraqi Board of Medical Specializations) further stated that the current research study does not require approval because it is a retrospective and has no intervention. Moreover, verbal consent was obtained from all participants in this study instead of written consent. For the minors, the patents and/or legal guardians’ consents were taken. This verbal consent approach was chosen for several reasons. First, many participants had low literacy levels, making written consent impractical and potentially exclusionary. Second, cultural sensitivities within the community associated signing documents with legal issues or distrust, so verbal agreements were more culturally appropriate and helped build rapport. Lastly, using verbal consent enhanced anonymity and confidentiality by avoiding written records that could compromise participants’ privacy, especially given the sensitive nature of the research topic. The Ethics Committee approved the verbal consent.

## Data Availability

The raw data is available at: Mohammad, N. (2024). Types of refractive errors in a sample of Iraqi children with Intermittent exotropia [Data set]. Zenodo. https://doi.org/10.5281/zenodo.13980859. ^26^ The project contains the following data:
•
Types_of_refractive_errors_in_a_sample_of_Iraqi_children_with_Intermittent.Updated 1.3.xlsx. Data are available under the terms of the Creative Commons Attribution 4.0 International license (CC-BY 4.0). No extended data is available.
