# Types of refractive errors in a sample of Iraqi children with Intermittent exotropia

**DOI:** 10.12688/f1000research.156932.5

**Published:** 2025-07-22

**Authors:** Najah K. Mohammad, Ibrahim Ali Rajab

**Affiliations:** 1 Department of Surgery, College of Medicine, University of Baghdad, Baghdad, Iraq

**Keywords:** Intermittent exotropia, emmetropia, myopia, refractive errors, pediatric strabismus.

## Abstract

**Background:**

One of the most common strabismus types in children is intermittent exotropia (IXE), which predominantly occurs in children between the ages of 2-4 years. It may affect visual development and often coexists with refractive errors. Unlike esotropia, which is typically associated with hyperopia, IXE demonstrates a more complex relationship with refractive errors. Recent studies suggest a possible link with myopia; however, this relationship remains unclear. This study will investigate the prevalence and distribution of refractive errors in children with IXE, challenging the traditionally held relationship between strabismus and hyperopia and its possible contribution to the development of myopia.

**Methods:**

In this cross-sectional retrospective study from August 2021 to December 2023, 179 patients diagnosed with IXE were recruited via an outpatient clinic in Najah Al-Quraishi, Baghdad, Iraq. The refractive errors were compared by autorefractometry and retinoscopy after cycloplegic dilation. Data analysis was conducted under the use of IBM SPSS V.26 for the determination of emmetropia, myopia, and hyperopia prevalence.

**Results:**

Among the 179 patients, emmetropia was the most commonly observed refractive status, present in 68 patients (38%). Low hyperopia and low myopia were also common, with 64 and 40, respectively. A limited number of patients had moderate/high myopia and moderate/high hyperopia; in detail, the prevalence was as follows: 5% of patients had moderate myopia, while 0.5% of patients suffered from high myopia; symmetrically, 5% were moderate to high hyperopia. From the data collected, a trend emerged for a low refractive error and symmetric refractive error in both eyes.

**Conclusion:**

Contrasted to prior conventional wisdom about refractive error in strabismus, it established the greater prevalence of emmetropia in patients with IXE. The findings call for specific management strategies to be applied in this population.

## Introduction

Intermittent exotropia (IXE) is an active type of strabismus that affects quite a large number of children. However, its prevalence might vary from one ethnic and age group to another. ^1, 2^ IXE is characterized by periodic outward deviation of one eye, which may disturb binocular vision in some patients. This has immense implications for visual development and the quality of life. The complicated relationship between IXE and refractive errors has been known, though not so much understood; according to some studies, different patterns of refractive anomalies could exist in these patients. ^1, 3^

Strabismus prevails in approximately 5%-8% of the general population; among these, exotropia ranks as one of the most common varieties, mainly between ages 2-4 years. ^1, 3^ IXE is probably considered the most tricky subset of exotropia in terms of coexistent refractive error and management. Whereas esotropia can be associated with plus, the relationship between exotropia and refractive errors is more complex. Of importance, a few studies have suggested a high prevalence of myopia among children with IXE, indicating that there might be an association between the two. However, most of these findings come from cross-sectional or descriptive data, and there is limited longitudinal evidence to establish causality. ^4^

Recent studies have begun to shed light on the complex interaction between IXT and myopia progression. A 12-month prospective study by Li et al. ^5^ found that children with basic IXT exhibited significantly greater myopic shift and axial elongation compared to non-strabismic peers, with the degree of progression correlating directly with the magnitude of exodeviation. This suggests that unstable binocular vision and increased accommodative demands may contribute to refractive changes in this population. Complementing these findings, Qian et al. ^6^ demonstrated that repeated low-level red-light therapy can effectively slow axial elongation in children with IXT, offering a promising intervention without worsening strabismus control. These emerging insights highlight the importance of considering both refractive and binocular factors in managing children with IXT.

In order to better understand the therapy and prognosis of intermittent exotropia, this study will look into the distribution of refractive errors in children and young people with the illness. It will also look for common refractive patterns.

## Methods

### Study design and setting

This is a cross-sectional retrospective study conducted at the corresponding author’s private ophthalmology clinic. The clinic serves a diverse population, primarily children from various walks of life suffering from ocular conditions, including intermittent exotropia. This being an observational study, there is no control. Hence, all eligible patients diagnosed with intermittent exotropia within the study period, ranging from August 2021 to December 2023, were included.

Patients were enrolled consecutively from the routine clinic visits. All subjects had been referred to the study from the clinics for clinical presentations of intermittent exotropia and, therefore, represented a homogenous population by diagnosis. No external advertisements for patient recruitment were utilized, and consecutive eligible patients presenting at the clinic were included, with further selection based only on exclusion criteria regarding previous ocular surgery or other types of strabismus.

Refractive errors were diagnosed by both auto-refractometry and retinoscopy following cycloplegia using 1% cyclopentolate. Three readings were taken for each eye. When discrepancies arose between the two methods, the result from retinoscopy was used for final classification due to its higher accuracy and clinical reliability in pediatric assessments. Testing was done 30 minutes after the final drop to ensure that complete cycloplegia had been achieved. All patients underwent three successive refractometry examinations on each eye to reduce variability. The amount of exotropia was measured by the prism cover test. All equipment was calibrated according to the manufacturer’s guidelines before use and checked regularly during the study for accuracy.

This study followed the STROBE checklist for cross-sectional studies. ^7^ The study design, setting, participants, variables, data sources, measurement techniques, and statistical methods are those in the list that apply and have been appropriately reported to ensure the reproducibility and transparency of the study. Therefore, a completed STROBE checklist is attached and provided to ensure verification of standards.

### Participants

This study only included patients whose inclusion criteria consisted of a proven clinical diagnosis of intermittent exotropia, ages from childhood to early adulthood, and those who gave their assent to participate in this research study. The exclusion criteria for this study include those patients with a history of ophthalmic surgery, all constant forms of strabismus, secondary exotropia ensuing from other eye pathology, and systemic conditions that affect visual function.

Squint division into two age groups, namely childhood below six years and school age six years or above, goes in line with the consideration of refractive errors. Both problems are of great educational and psychosocial concern to these age groups, especially since the starting age for school is six years old in Iraq. ^8, 9^

### Sample size determination

The total number of participants involved in this study was calculated according to the number of patients who presented with intermittent exotropia in the consultation at the outpatient clinic from August 2021 to December 2023. It is an exhaustive sample of all eligible patients who visited with a diagnosis of intermittent exotropia from August 2021 to December 2023. 179 patients were included to be eligible out of 195, and the rest were excluded mainly for insufficient data.

### Data collection

Refractive status was classified based on spherical equivalent refraction (SER), calculated by adding half the cylindrical power to the spherical power. Patients were categorized as emmetropic if their SER fell between -0.50 and +0.50 diopters. Myopia was defined as an SER less than -0.50 diopters and further subdivided into low myopia (more than -3.00 D and up to -0.50 D), moderate myopia (more than -6.00 D and up to -3.00 D), and high myopia (equal to or less than -6.00 D). Hyperopia was defined as an SER greater than +0.50 diopters and was classified into low hyperopia (up to +3.00 D) and moderate to high hyperopia (greater than +3.00 D). Children with astigmatism were not excluded; instead, their classification into myopia, hyperopia, or emmetropia was based on the SER derived from both spherical and cylindrical values.

Still, the divisions of hypermetropia are not distinct in the readings except that follow a similar rating scale depending on the magnitude of refractive error. ^8^ Written consent was waived since the data in this study were retrospectively analyzed from medical records, and no direct contact with the patients was required to collect any data. However, in regard to further data collection from the patients during follow-up visits and that of minors, verbal consent was obtained, approved by the Ethics Committee. In the participants below 18 years of age, consent was obtained from their parents or guardians. Verbal consent was documented due to cultural and logistical barriers that made obtaining written consent impractical for some participants. Age was divided into two groups: those aged 6 years or older, and those younger than 6 years.

### Statistical analysis

Data analysis was done using IBM SPSS V.26, where descriptive statistics summarized data and tabulated results in accounts and percentages of each category of refractive error. Another level summary of analysis was undertaken by calculating the mean for spherical equivalent refraction (SER) correction outcome of either eye. These were also tabulated and interpreted to make sense of the refractive trends in this population of patients.

The data were checked for completeness and accuracy before statistical analysis; partial refraction records and those values that did not logically fit the established norms, after cross-checking with the patients’ records, were screened out. The outliers were defined as those values that lay beyond three standard deviations from the mean of the distribution and were reviewed for possible recording errors. These were included if clinically relevant, as this reflected extreme cases of refractive errors. Sensitivity analyses excluding these outliers were conducted to see the robustness of the results. Overall analysis was conducted using IBM SPSS V.26, with descriptive statistics conducted to summarize patient demographics and refractive error distributions.

Normality was checked by the Shapiro-Wilk test to decide which set of statistics tests should be applied. Parametric tests were conducted for normally distributed data; otherwise, non-parametric tests were conducted. Outliers, defined as values more than three standard deviations away from the mean, were removed from analysis after confirmation of their non-representative nature. Descriptive statistics summarized data by using means and standard deviations for continuous variables and percentages for categorical variables. All statistical analyses were performed using IBM SPSS V.26.

## Results

A total of 179 patients with intermittent exotropia were included in this study. Of these, 85 (47.5%) were male, while 67 (37.4%) were children, and 112 (62.6%) were young students. The demographics of the study participants are summarized in Table 1.

**Table 1.** Demographics of study participants.

Demographic variable	Category	Count	Percentage (%)
Gender	Male	85	47.5
	Female	94	52.5
Age group	Children (below 6)	67	37.4
	Young students (6 and above)	112	62.6
Total		179	100

### Prevalence of refractive errors

**Figure 1.**
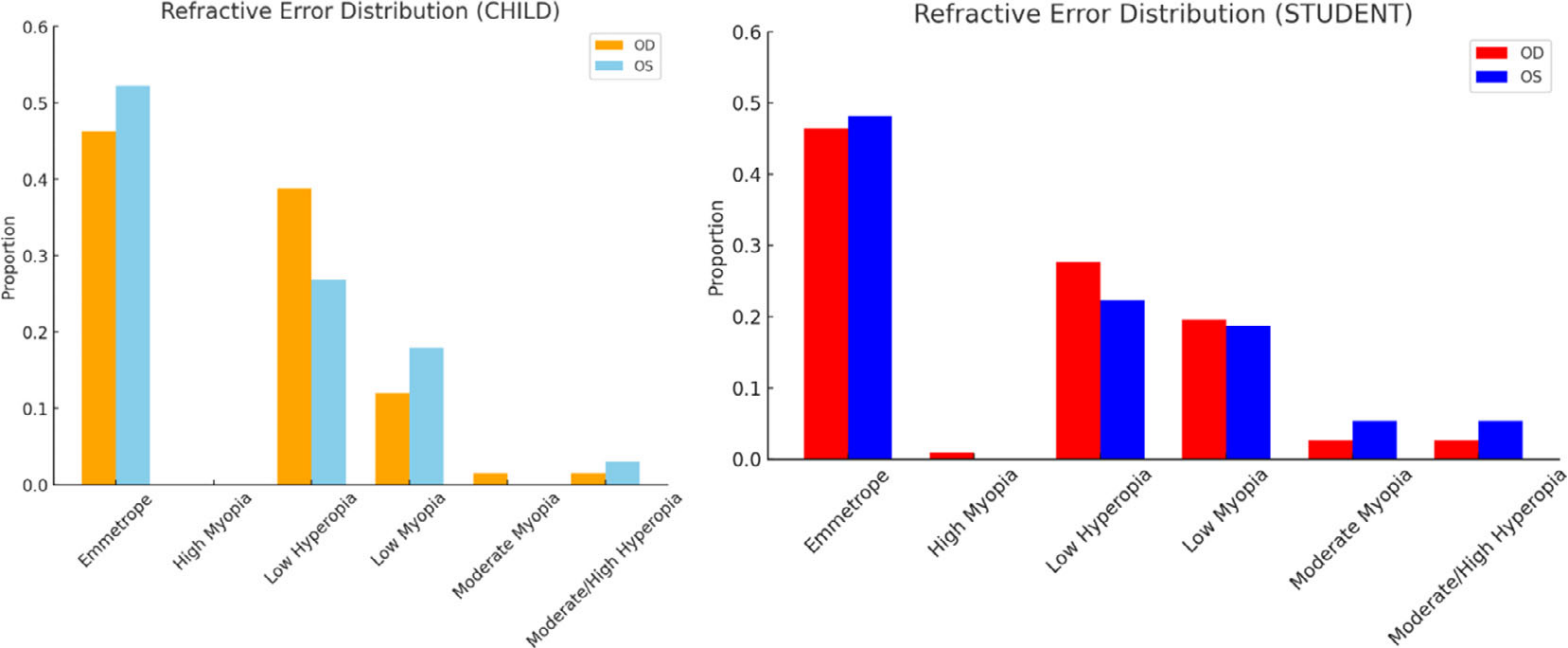
Heatmap of refractive errors between the two eyes.

Emmetropia was the most common refractive status, observed in 68 patients (38%). Low hyperopia and low myopia followed, affecting 64 (35.7%) and 40 (22.3%) patients, respectively. Moderate myopia was present in 9 patients (5%), high myopia in 1 patient (0.5%), and moderate to high hyperopia in 9 patients (5%) ( Figure 1).

### Symmetry in refractive errors

**Figure 2.**
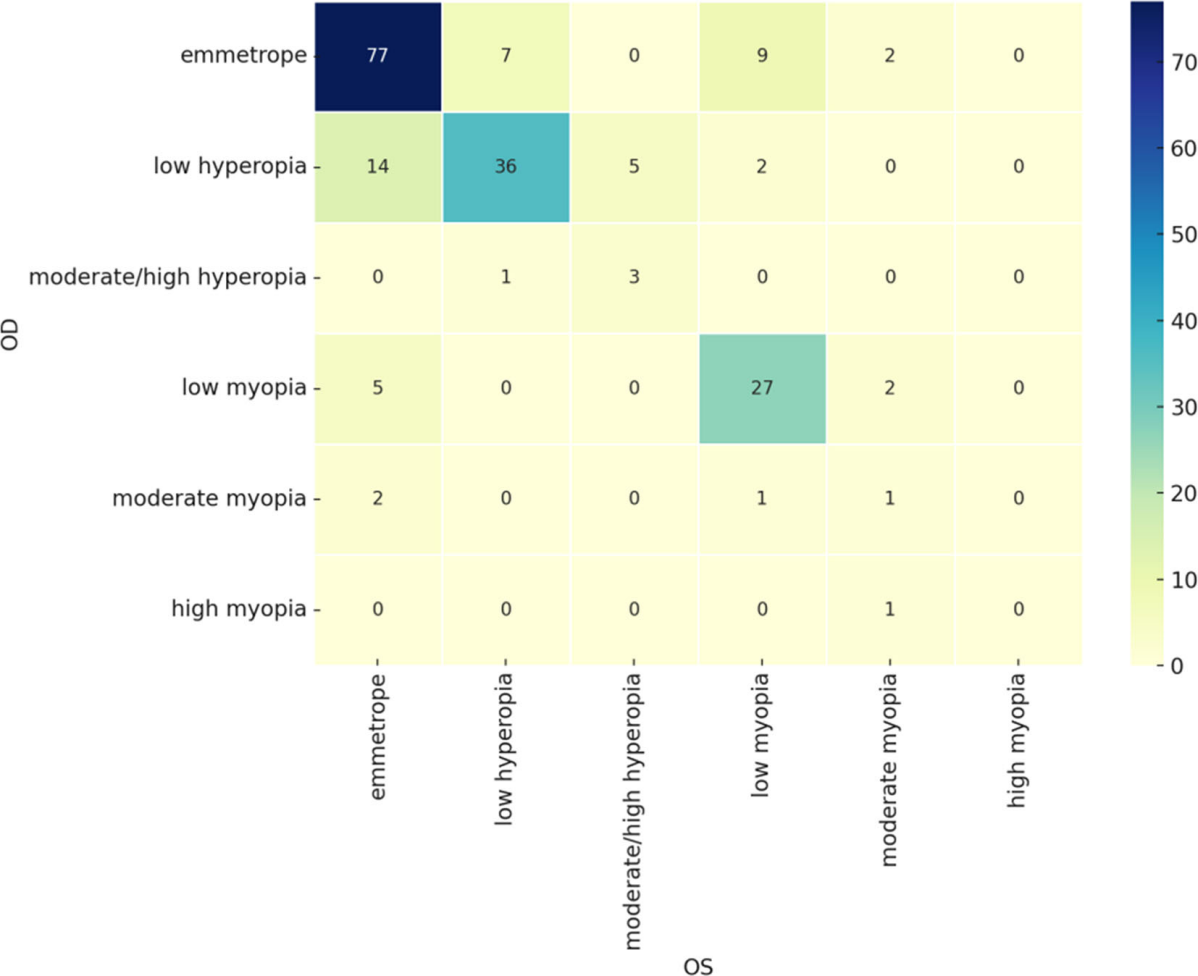
Confusion matrix for the refractive status of right and left eyes.

A cross-tabulation of refractive errors between the two eyes indicated a high degree of symmetry. Most patients exhibited very similar refractive classifications in both eyes, particularly those who were emmetropic or had low myopia, as illustrated in the confusion matrix ( Figure 2). This bilateral consistency suggests a strong underlying correlation in refractive status, even though no formal statistical test was performed. While some asymmetry was observed in cases with moderate to high refractive errors, these represented a small minority of the sample was initially applied, the distribution suggests strong bilateral consistency.

### Detailed refractive status analysis

Emmetropia showed a mean spherical equivalence of 0.118 in the right eye and 0.175 for the left eye. Low myopia had a mean spherical equivalence of -1.83 and -1.68 for right and left eye respectively, while moderate myopia showed a mean spherical equivalence of -3.59 for OD and -3.59 for OS. High myopia was rare, with only one case, showing a mean spherical equivalence of -6.25 for OD and not applicable for OS, details are shown in Table 2. The relationship between gender and age with different refactive status is shown in Table 3 with no significant association; p-value 0.1 and 0.3, respectively.

**Table 2.** Mean spherical equivalent refraction values (in diopters) for different refractive error categories in patients with intermittent exotropia, comparing the right eye (OD) and left eye (OS).

Category	Mean spherical equivalence OD	Mean spherical equivalence OS
Emmetropia	0.118	0.175
Low Myopia	-1.83	-1.68
Moderate Myopia	-3.59	-3.59
Low Hyperopia	1.63	1.52
Moderate/High Hyperopia	4.56	4.06

**Table 3.** The association between gender, age, and refractive status.

		OD		OS	
Refractive status	Gender	Counts	% of Total	Counts	% of Total
emmetrope	Female	40	42.55	44	46.80
	Male	43	50.58	45	52.94
high myopia	Female	1	1.06	0	0
	Male	0	0	0	0
low hyperopia	Female	29	30.85	23	24.46
	Male	28	32.94	20	23.52
low myopia	Female	21	22.34	20	21.27
	Male	9	10.58	13	15.29
moderate myopia	Female	3	3.19	4	4.25
	Male	1	1.17	2	2.35
moderate/high hyperopia	Female	0	0	3	3.19
	Male	4	4.70	5	5.88
**Age groups**					
emmetrope	≥6	52	62.7%	54	48.21%
	<6	31	37.35%	35	52.23%
high myopia	≥6	1	0.89%	0	0
	<6	0	0	0	0
low hyperopia	≥6	31	27.67%	25	22.32%
	<6	26	38.80%	18	26.86%
low myopia	≥6	22	19.64%	21	18.75%
	<6	8	11.94%	12	17.91%
moderate myopia	≥6	3	2.67%	6	5.35%
	<6	1	1.49%	0	0
moderate/high hyperopia	≥6	3	2.67%	6	5.35%
	<6	1	1.49%	2	2.98%

## Discussion

In our cohort, emmetropia was observed in 38% of children with IXT, a prevalence notably higher than that typically found in esotropic populations and comparable to some non-strabismic groups. In the Baltimore Pediatric Eye Disease Study, emmetropia (defined as SE between −1.00 and +1.00 D) was reported in 35.6% of White children and 58.0% of African-American children aged 6–71 months. ^11^ Similarly, Birch et al. ^12^ found that in children with infantile esotropia, the typical early-life emmetropization seen in normative cohorts was absent; these children maintained moderate hyperopia through age 7 and only experienced a refractive decline beginning around age 8. These data support the view that intermittent exotropia may represent a distinct refractive phenotype, exhibiting a higher prevalence of emmetropia than esotropia and possibly approximating that seen in certain non-strabismic populations. 

Probably the most striking fact brought out by this study of intermittent exotropia—an eye misalignment characterized by outward deviation—is the remarkably high prevalence of emmetropia, wherein fully 50% of the patient sample showed no refractive error. ^13^ This rate not only forms the bulk of the refractive statuses but contrasts significantly with the expected rates for myopia and hypermetropia traditionally associated with strabismic conditions. ^13^ This high quantity of emmetropia strongly suggests that intermittent exotropia does not follow the usual patterns of refractive error seen in most other strabismus.

This study ranges from 20 to 23% in terms of the prevalence of myopia among the cohort, standing in striking contrast to the 55.5% of myopia prevalence in the cohort studied by Robaei et al. ^14^ This discrepancy may reflect demographic, genetic, or environmental differences between the populations or, perhaps more likely, differing criteria for classifying myopia. Our study corroborates findings from population-based research, which identified refractive errors as significant contributors to exotropia. Notably, Robaei et al. ^14^ also found markedly lower rates of emmetropia and hyperopia among exotropia patients—only 7.7% were hyperopic (≥ +2.00 D), and emmetropic eyes were underrepresented—whereas our cohort demonstrated a higher prevalence of emmetropia (38%) and a more balanced refractive distribution. Specifically, the multi-ethnic cohort study emphasized the association between higher degrees of astigmatism (≥1.5 D) and anisometropia with the development of exotropia. These risk factors parallel the refractive trends observed in our population, highlighting the importance of addressing underlying refractive errors in managing exotropia effectively. ^15^

Our research further elaborates on the relationship between hyperopia and intermittent exotropia. While this association has been reported constantly, our findings give further sophistication to this understanding in pointing out that hyperopia is not as frequent as emmetropia or myopia within this type of strabismic condition. This makes us think about thought whether the refractive error profile for intermittent exotropia might differ from other strabismic disorders in which hyperopia shows up more frequently. ^16– 18^

This study highlights the observation that while myopia was the least common refractive state among children diagnosed with intermittent exotropia, its prevalence was higher in older children compared to younger ones. This trend may suggest a possible age-related myopic shift in IXT patients, but our findings do not support a direct association. The observed differences may reflect underlying factors such as increased accommodative demand or genetic predisposition, which warrant further longitudinal investigation. ^19^

Although intriguing, it remains speculative to suggest from the current data that intermittent exotropia is an etiological factor in the development of myopia. One hypothesis is that the increased accommodative effort required to maintain binocular alignment in IXT may contribute to myopic progression. This is consistent with previous studies showing that reducing accommodative demand may slow the progression of intermediate myopia. However, this theory remains unconfirmed and should be interpreted with caution. ^20^^,^^21^

Given our findings, we suggest that management of intermittent exotropia should consider the refractive profile, particularly the high prevalence of emmetropia and low hyperopia. In such cases, conservative monitoring may be sufficient. While myopia was less common, its presence in older children may support careful follow-up to monitor for progression. Although we did not present interventional data, the discussion of therapeutic strategies, such as reducing accommodative strain, remains relevant and is supported by existing literature. ^22^^,^^23^ However, we acknowledge that further studies are needed to evaluate the impact of such interventions in this specific population.

These findings are in agreement with reports of previous studies that intermittent exotropia may have a refractive error profile different from other forms of strabismus. For example, the relatively high prevalence of emmetropia and low hyperopia in our cohort, compared to the types usually observed among esotropic patients, underlines this distinct distribution of refractive errors and further justifies the need for diagnostic and therapeutic strategies specific to intermittent exotropia. ^24, 25^ This differentiation of treatment strategies is essential for the designation of intermittent exotropia as an independent clinical entity, outlining its characteristics of refraction. ^26^

Our study also opens the window for further understanding of how these refractive error patterns may influence long-term visual outcomes. The relative prevalence of high myopia and hyperopia may be low, and the prognosis for intermittent exotropia might turn out to be relatively favorable when compared to those with constant esotropia or exotropia if timely and proper measures are implemented. ^27^ This fact heightens concern for regular ophthalmologic follow-up examination and early corrective measures to avoid the progression of refractive errors and achieve optimum visual development. ^28^ Longitudinal studies in the future must be directed toward understanding the progression of refractive error in intermittent exotropia and setting up evidence-based guidelines for its management. ^29, 30^

The current study offers two significant insights into the research on the distribution of refractive errors in patients with intermittent exotropia and the possible association between strabismus and the development of myopia. Our results point toward possible associations of intermittent exotropia with myopic trends. However, no causation can be established since this is a cross-sectional study. These noted patterns indicate the need for more customized treatment approaches that must involve strabismus and any refractive errors related to it. However, due to the lack of longitudinal data defining the temporal development of refractive errors relative to the onset and progression of intermittent exotropia, we cannot confirm whether any observed relationships are causative. Further studies with a longitudinal design will be necessary for elucidating such dynamics further and refining management strategies. The high prevalence of emmetropia in intermittent exotropia, as seen in this study, needs to be put into the proper perspective of refractive error distribution in populations with and without strabismus. Such a comparison is important to establish whether this prevalence represents a particular characteristic of IXT or part of a general trend seen in normal binocular vision. Thus, on examining the trends of refractive errors in children with intermittent exotropia, several points of interest reflect deficits in prior literature regarding diagnosis or research methodology. Since then, these different types of strabismus have not been analyzed carefully about their typical refractory predisposition either theoretically or experimentally to enable good estimations between them. Most had research in homogeneous populations only; however, sometimes even sample sizes proved limited to achieve strong, statistical generalization. Longitudinal data on causality in the relationship between intermittent exotropia and the development of myopia are lacking. This study specifically addresses these gaps by focusing on intermittent exotropia within a large, diverse pediatric population; future research with longitudinal designs is imperative for confirmation and expansion of these findings.

However, the limitations of our study merit mention. One major limitation in the drawing of solid conclusions regarding the causality of intermittent exotropia and refractive errors is not having of a control group of non-spectacle-wearing subjects. Second, there is still missing longitudinal data defining the temporal development of refractive errors relative to the onset and progression of intermittent exotropia. Although our retrospective design did not allow for a normal binocular vision control group, such a comparison would indeed be very valuable in the future, especially in the prospective studies that would like to tease out whether this prevalence is a unique feature of intermittent exotropia or rather part of the broader pattern of refractive error. One notable limitation of this study is the use of a clinic-based sample, which may not fully represent the general population. Individuals presenting to ophthalmology clinics may differ in important ways compared to the broader community. This paper thus sheds light on the refractive landscape of intermittent exotropia. Still, it makes an even stronger case for other and more comprehensive studies to tease out complicated interactions between refractive errors and this common form of strabismus.

## Conclusions

Our study showed that, contrary to the traditionally expected predominance of myopia and hyperopia in strabismus, the most frequent refractive status in intermittent exotropia patients was emmetropia, followed by hypermetropia. While myopia was the least common, its presence in older children warrants further investigation into potential age-related trends. These findings highlight the need for longitudinal studies to explore how refractive profiles evolve in children with intermittent exotropia.

## Ethics and consent

The Ethics Committee (Iraqi Board of Medical Specializations) further stated that the current research study does not require approval because it is a retrospective and has no intervention. Moreover, verbal consent was obtained from all participants in this study instead of written consent. For the minors, the parents and/or legal guardians’ consents were taken. This verbal consent approach was chosen for several reasons. First, many participants had low literacy levels, making written consent impractical and potentially exclusionary. Second, cultural sensitivities within the community associated signing documents with legal issues or distrust, so verbal agreements were more culturally appropriate and helped build rapport. Lastly, using verbal consent enhanced anonymity and confidentiality by avoiding written records that could compromise participants’ privacy, especially given the sensitive nature of the research topic. The Ethics Committee approved the verbal consent.

## Data Availability

The raw data is available at: Mohammad, N. (2024). Types of refractive errors in a sample of Iraqi children with Intermittent exotropia [Data set]. Zenodo. https://doi.org/10.5281/zenodo.13980859. ^31^ The project contains the following data:
•
Types_of_refractive_errors_in_a_sample_of_Iraqi_children_with_Intermittent.Updated 1.3.xlsx. Data are available under the terms of the Creative Commons Attribution 4.0 International license (CC-BY 4.0). No extended data is available.
