# Supplementary material for: Types of refractive errors in a sample of Iraqi children with Intermittent exotropia
Source: F1000Res. 2025 Jul 22;13:1318. Originally published 2024 Nov 5. [Version 5] doi: 10.12688/f1000research.156932.5 (PMC12457882; doi:10.12688/f1000research.156932.5)
Supplement: Peer review history

# Reviewer report: Reviewer response for version 5

**DOI:** 10.5256/f1000research.183024.r407311

**Published:** 2025-09-23

**Authors:** Hector Santiago

**Affiliations:** 1 Inter American University of Puerto Rico, Bayamón, USA

The study addresses an understudied population of Iraqi children with IXE. The sample size seems adequate.

 We respect the decision of the Iraqi Ethics Board. However, most ethics boards require approval of patients for the use of retrospective clinical data for research: If patients give no explicit permission, clinical data can’t be used. Also, most ethics boards require written consent or, for small children, assent.

 The main weakness of the paper is the lack of an age-matched group of Iraqi children without strabismus for comparison. We don’t know if the distribution of refractive errors is significantly different from that of the age-matched group. Also, as all clinic-based samples, it may not represent the general population of patients with the condition.

 1. Which data was normally distributed? Which was not? Explain in each section which statistical tests were conducted.

 2. For symmetry of refractive error, you can calculate the Pearson correlation of the SE OD and SE OS. If the data is non-normal, one can use Spearman’s rank or Kendall’s tau.

 3. Present a table of the distribution of the sample by age and sex. Not clear what the age range (youngest and oldest) is.

 4. It should say: “ esotropia is associated with hyperopia”  (not associated with plus)

 5. The method section is unclear. Did all patients have cycloplegia, followed by autorefractor, and then retinoscopy? How many drops of cyclopentolate 1% per eye?

Is the work clearly and accurately presented and does it cite the current literature?

Partly

If applicable, is the statistical analysis and its interpretation appropriate?

Partly

Are all the source data underlying the results available to ensure full reproducibility?

No

Is the study design appropriate and is the work technically sound?

Partly

Are the conclusions drawn adequately supported by the results?

Partly

Are sufficient details of methods and analysis provided to allow replication by others?

Partly

Reviewer Expertise:

Prevalence of refractive error and visual impairment

I confirm that I have read this submission and believe that I have an appropriate level of expertise to confirm that it is of an acceptable scientific standard, however I have significant reservations, as outlined above.

# Reviewer report: Reviewer response for version 4

**DOI:** 10.5256/f1000research.180118.r382155

**Published:** 2025-05-14

**Authors:** Janos Nemeth

**Affiliations:** 1 Semmelweis University, Budapest, Budapest, Hungary

In general, the study may be of interest to the scientific reader community because it is based on a large population cohort of individuals with intermittent exotropia (IXT) and studied the distribution of their refractive states. However, the analysis, presentation and discussion of the results would need further development and clarifications. The discussion need to be far shortened and concentrated to the results. The manuscript needs language and logistic corrections by a native English expert, due to several unclear statements in the abstract and in the text of the article, like e.g. the last sentence of the results, or in the Introduction: „Whereas esotropia can be associated with plus, …” – What „plus” means here?

 Detailed comments.

 I do not agree with the unsupported last two statements in the Background (that „… intermittent exotropia might … lead to myopia”). The mention of a causative relationship is too strong statement based on the literature list of the manuscript version 4. However, there are some new publications which might support the authors. Please, cite also these publications: 

 (refer to 1 ).

 ( refer to 2 )

 Please, show the age range of the study participants in the abstract.

 In some places the authors mention only auto-refractometry and in some other places both auto-refractometry and retinoscopy for determination of the refraction. What is the reality? How the decision was made if auto-refractometry and retinoscopy presented different results?

 Definitions of refractive errors are presented twice in Paragraph 1 of Data collection and slightly differently (see e.g. low myopia: <-0.5D - -3.0D vs <-0.75D - -3.0D). 

 Where children with astigmatism in the studied groups? How did the authors classify them?

 Please, specify and describe definitely the ages (range, mean, SD) of the participants in the different groups at the date of the cross-sectional examination.

 Figure 1. Due to the different number of participants in the two groups, it would be advisable to show their refractive status in two separate sub-figures (one for children and the other for young students) and in percentages instead of the number of persons.

 The mean spherical equivalents (in the text and also in Table 2) need to be shown with decimals, one or two decimals depending on the accuracy of the refraction measurements. E.g. if the refraction is determined by one decimal accuracy, two decimals need to be exhibited when showing the mean value.

 Table 1 and Table 3 shows age limits between the 2 subgroups. Less than 6 years of age or more than 6 years of age. What about those who were exactly 6 years old? Please, explain the definitions of age subgroups also in the text of the Methods.  

 The Count numbers are false in Table 3 and they are different than in Table 1. In Table 3 there are 99 females and 96 males for the right eye and 147 females and 146 males for the left eye! While in Table 1 only 83 females and 96 males are mentioned. In Table 3 there are 67 persons below the age of 6 years and 128 above for the right eye but for the left eye these numbers are quite higher: 191 and 102 respectively. Both are different than in Table 1. 

 The percentage calculations are wrong in Table 3. because of the number of persons in the two gender groups and also in the two age groups are not equal (68 vs 128 persons), therefore the % calculations have to perform for the two subgroups independently.  

 The authors missed to compare the distributions of the refraction states between the two age groups. They did not mention that in the older group, the percentage of emmetropia is higher (by 8.1%), myopia is far higher (by 74.5%) and hyperopia is far lower (by -34.1%) than in the younger age group (based on the data of the right eye in Table 3 which count numbers are close to the count numbers in Table 1).  

 Discussion

 First paragraph. Please, present the results, (the found percent of emmetropia,) in the context of the literature data of similar age groups of non-strabismus children and that of esotropia children and exotropia children.

 The best way would be if the authors would have own controls for children with non-strabismus and esotropia. However, as the authors have no such controls, the data might be compared to literature data of children with similar ages and ethnicity. Only after such comparisons might be made a supported statement that the found percentage of emmentropia is higher, similar or lower than in the corresponding literature. (It is scientifically not enough and acceptable to relate the findings only to “prior conventional wisdom” as the authors mentioned.) 

 In the comparison with Robaei et al data, there might be mentioned some other important differences apart from myopia, namely: Robaei et al found far less emmetrops and hyperops among exotropia patients. 

 In Paragraph 4 in the Discussion, I do not agree with the authors statement that they observed a myopia prevalence which might suggest a possible association with IXT because their data presented in the manuscript do not support this. Among the refractive state, emmetropia and then phyperopia are the most prominent and myopia is only the last most frequent. However, I would agree that the prevalence of myopia in the older age group is far higher than in the younger age group which might be an indirect sign of a myopic shift with age in IXT children.

 As the authors presented no any longitudional data, it would be advisable not to present long discussion and conclusions on the existence of myopia in children with exotropia and on their relationship, as these are unsupported conclusions. Please, shorten this part of the discussion extensively.

 As author presented no data on the effect of therapy, the discussion about it is unsupported by the authors own data and therefore it is suggested to delete.

 The last sentence in the final conclusion is also unsupported, so need to modify the statement.

Is the work clearly and accurately presented and does it cite the current literature?

Partly

If applicable, is the statistical analysis and its interpretation appropriate?

Partly

Are all the source data underlying the results available to ensure full reproducibility?

Partly

Is the study design appropriate and is the work technically sound?

Yes

Are the conclusions drawn adequately supported by the results?

Partly

Are sufficient details of methods and analysis provided to allow replication by others?

Partly

Reviewer Expertise:

Refractive errors, epidemiology of eye diseases, myopia prevention and management, cornea, glaucoma, blindness prevention, blindness prevalence.

I confirm that I have read this submission and believe that I have an appropriate level of expertise to confirm that it is of an acceptable scientific standard, however I have significant reservations, as outlined above.

## References

[1] : Effects of basic type of intermittent exotropia on myopic shift in children: a 12-month observational study. *Front Pediatr* .2024;12: 10.3389/fped.2024.1513062 1513062 10.3389/fped.2024.1513062 39834488 PMC11743707

[2] : Effects of repeated low-level red-light therapy on myopia control in children with intermittent exotropia. *Photodiagnosis Photodyn Ther* .2025;52: 10.1016/j.pdpdt.2025.104526 104526 10.1016/j.pdpdt.2025.104526 39978611

# Response

**Published:** 2025-05-28

**Authors:** Najah Mohammad

**Affiliations:** Department of Surgery, University of Baghdad, Baghdad, Baghdad Governorate, Iraq

We sincerely thank the editorial team and reviewer for their time, constructive feedback, and commitment to improving the quality of our manuscript.

**In general, the study may be of interest to the scientific reader community because it is based on a large population cohort of individuals with intermittent exotropia (IXT) and studied the distribution of their refractive states. However, the analysis, presentation and discussion of the results would need further development and clarifications. The discussion needs to be far shortened and concentrated on the results. The manuscript needs language and logistic corrections by a native English expert, due to several unclear statements in the abstract and in the text of the article, like e.g. the last sentence of the results, or in the Introduction: „Whereas esotropia can be associated with plus, …” – What „plus” means here?**

**Response: **Thank you for your constructive feedback. We will revise the manuscript thoroughly for clarity and conciseness, particularly in the Discussion section, and will seek assistance from an expert English speaker to improve language quality. The term "plus" in the Introduction will be replaced with "hyperopia" to avoid ambiguity.

**Detailed comments.**

**I do not agree with the unsupported last two statements in the Background (that „… intermittent exotropia might … lead to myopia”). The mention of a causative relationship is too strong statement based on the literature list of the manuscript version 4. However, there are some new publications which might support the authors. Please, cite also these publications:  (refer to 1 ); ( refer to 2 )**

**Response: **Thank you for your valuable comment. We agree that the original statement may have implied causation too strongly. We have revised the wording to reflect an association rather than causality and have incorporated the suggested recent references to support the manuscript more appropriately.

**Please, show the age range of the study participants in the abstract.**

**Response: **The study included patients based on predefined age groups, as outlined in the methodology section, without specifying their exact ages.

**In some places the authors mention only auto-refractometry and in some other places both auto-refractometry and retinoscopy for determination of the refraction. What is the reality? How the decision was made if auto-refractometry and retinoscopy presented different results?**

**Response: **Thank you for highlighting this inconsistency. We confirm that both auto-refractometry and retinoscopy were performed for all participants. In cases of discrepancy, retinoscopy was considered the final and more accurate method for classification. We have revised the manuscript to reflect this clearly and consistently.

**Definitions of refractive errors are presented twice in Paragraph 1 of Data collection and slightly differently (see e.g. low myopia: <-0.5D - -3.0D vs <-0.75D - -3.0D).**

**Where children with astigmatism in the studied groups? How did the authors classify them?**

**Response: **Thank you for your careful observation. We have removed the duplicate and inconsistent definitions and unified the classification based on spherical equivalent refraction. Children with astigmatism were included in the analysis and classified according to their spherical equivalent values. The methodology section has been revised accordingly for clarity.

**Please, specify and describe definitely the ages (range, mean, SD) of the participants in the different groups at the date of the cross-sectional examination.**

**Response: **The study included patients based on predefined age groups, as outlined in the methodology section, without specifying their exact ages.

**Figure 1. Due to the different number of participants in the two groups, it would be advisable to show their refractive status in two separate sub-figures (one for children and the other for young students) and in percentages instead of the number of persons.**

**Response: **The figure was divided into two subfigures based on proportional distribution, as recommended.

**The mean spherical equivalents (in the text and also in Table 2) need to be shown with decimals, one or two decimals depending on the accuracy of the refraction measurements. E.g. if the refraction is determined by one decimal accuracy, two decimals need to be exhibited when showing the mean value.**

**Response: **Two decimal places were used to present the mean spherical equivalent for greater precision.

**Table 1 and Table 3 shows age limits between the 2 subgroups. Less than 6 years of age or more than 6 years of age. What about those who were exactly 6 years old? Please, explain the definitions of age subgroups also in the text of the Methods.**

**Response:   **The exact definition of age groups was demonstrated in the methods.

**The Count numbers are false in Table 3 and they are different than in Table 1. In Table 3 there are 99 females and 96 males for the right eye and 147 females and 146 males for the left eye! While in Table 1 only 83 females and 96 males are mentioned. In Table 3 there are 67 persons below the age of 6 years and 128 above for the right eye but for the left eye these numbers are quite higher: 191 and 102 respectively. Both are different than in Table 1.**

**Response: **The numbers and percentages in table 3 have been thoroughly rechecked, and the errors have been corrected accordingly.

**The percentage calculations are wrong in Table 3. because of the number of persons in the two gender groups and also in the two age groups are not equal (68 vs 128 persons), therefore the % calculations have to perform for the two subgroups independently.**

**Response: **The numbers and percentage calculations have been reviewed, and the necessary corrections were made to reflect the correct subgroup proportions.

****

**The authors missed to compare the distributions of the refraction states between the two age groups. They did not mention that in the older group, the percentage of emmetropia is higher (by 8.1%), myopia is far higher (by 74.5%) and hyperopia is far lower (by -34.1%) than in the younger age group (based on the data of the right eye in Table 3 which count numbers are close to the count numbers in Table 1).**

**Response: **The authors compared the distribution of refractive states across different age groups and found no statistically significant differences between them.

****

**Discussion**

**First paragraph. Please, present the results, (the found percent of emmetropia,) in the context of the literature data of similar age groups of non-strabismus children and that of esotropia children and exotropia children. The best way would be if the authors would have own controls for children with non-strabismus and esotropia. However, as the authors have no such controls, the data might be compared to literature data of children with similar ages and ethnicity. Only after such comparisons might be made a supported statement that the found percentage of emmentropia is higher, similar or lower than in the corresponding literature. (It is scientifically not enough and acceptable to relate the findings only to “prior conventional wisdom” as the authors mentioned.)**

**Response: **Thank you for this insightful comment. In response, we have added a comparative analysis of the emmetropia prevalence in our cohort against literature data to provide clearer context and highlight the distinct refractive error profile in intermittent exotropia.

**In the comparison with Robaei et al data, there might be mentioned some other important differences apart from myopia, namely: Robaei et al found far less emmetrops and hyperops among exotropia patients.**

**Response: **Thank you for the valuable suggestion. We have revised the paragraph to include the lower prevalence of emmetropia and hyperopia among exotropia patients in the Robaei et al. study, providing a more comprehensive comparison.

**In Paragraph 4 in the Discussion, I do not agree with the authors statement that they observed a myopia prevalence which might suggest a possible association with IXT because their data presented in the manuscript do not support this. Among the refractive state, emmetropia and then phyperopia are the most prominent and myopia is only the last most frequent. However, I would agree that the prevalence of myopia in the older age group is far higher than in the younger age group which might be an indirect sign of a myopic shift with age in IXT children. As the authors presented no any longitudional data, it would be advisable not to present long discussion and conclusions on the existence of myopia in children with exotropia and on their relationship, as these are unsupported conclusions. Please, shorten this part of the discussion extensively.**

**Response: **Thank you for your observation. We have revised the paragraph to clarify that while myopia was the least frequent overall, its increased prevalence in the older age group may reflect a potential age-related myopic shift in IXT, rather than a primary association.

**As author presented no data on the effect of therapy, the discussion about it is unsupported by the authors own data and therefore it is suggested to delete.**

**Response: **Thank you for your comment. We agree that the original discussion extended beyond the data presented. We have now shortened this section to avoid unsupported conclusions

**The last sentence in the final conclusion is also unsupported, so need to modify the statement.**

**Response: **Thank you for the comment. We have revised the conclusion to better reflect the descriptive nature of our results and to avoid unsupported causal inferences.

# Reviewer report: Reviewer response for version 4

**DOI:** 10.5256/f1000research.180118.r376737

**Published:** 2025-04-11

**Authors:** Dr. Ankit Varshney

**Affiliations:** 1 Shree Bharatimaiya College of Optometry and Physiotherapy, Veer Narmad South Gujarat University, Surat, Gujarat, India

Everything is fine, no further comment.

Is the work clearly and accurately presented and does it cite the current literature?

Yes

If applicable, is the statistical analysis and its interpretation appropriate?

Partly

Are all the source data underlying the results available to ensure full reproducibility?

Yes

Is the study design appropriate and is the work technically sound?

Partly

Are the conclusions drawn adequately supported by the results?

Partly

Are sufficient details of methods and analysis provided to allow replication by others?

Partly

Reviewer Expertise:

Dispensing Optics, Contact Lens, Orthoptics, Low Vision, Anterior Segment, Posterior Segment, Refractive Error

I confirm that I have read this submission and believe that I have an appropriate level of expertise to confirm that it is of an acceptable scientific standard.

# Reviewer report: Reviewer response for version 3

**DOI:** 10.5256/f1000research.176025.r370659

**Published:** 2025-03-18

**Authors:** Dr. Ankit Varshney

**Affiliations:** 1 Shree Bharatimaiya College of Optometry and Physiotherapy, Veer Narmad South Gujarat University, Surat, Gujarat, India

**Please find the Full Report on Manuscript Review:**

 The article titled *"Types of Refractive Errors in a Sample of Iraqi Children with Intermittent Exotropia"* investigates the prevalence and distribution of refractive errors in children diagnosed with intermittent exotropia (IXT). The study is a **cross-sectional retrospective analysis** of 179 patients recruited from an ophthalmology clinic in Baghdad, Iraq. Refractive errors were measured using **autorefractometry and retinoscopy after cycloplegia**, and data were analyzed using **IBM SPSS V.26**.

 Key findings include:

- **Emmetropia was the most common refractive status (38%)**, followed by low hyperopia (35.7%) and low myopia (22.3%).
- Moderate-to-high myopia and hyperopia were **less prevalent (5% each)**.
- **Symmetry in refractive errors between both eyes** was observed in most cases.
- The authors suggest that **intermittent exotropia may be associated with myopia development**, but acknowledge the need for longitudinal studies.

While the study contributes valuable data to the field of pediatric ophthalmology, there are several **methodological and interpretational concerns** that need to be addressed before the article can be considered scientifically sound.

**Strengths:**

- The manuscript is well-structured and presents its findings in a logical format.
- The **revised version** of the article has improved clarity and removed some misleading terminology (e.g., replacing “incidence” with “prevalence”).
- The discussion section provides **clinical implications** based on the findings.
- **The use of cycloplegic refraction** is a standard and reliable method for refractive error assessment.
- The **STROBE checklist** is followed, ensuring methodological transparency.
- **Outlier analysis and normality testing (Shapiro-Wilk test)** strengthen the statistical approach.
- Clear descriptions of **data collection procedures and statistical software**.
- The **public availability of raw data on Zenodo** supports transparency.
- Use of **SPSS V.26**, normality tests, and sensitivity analysis strengthen the methodology.
- Clear **prevalence reporting** of refractive error subtypes.
- The dataset is **publicly available on Zenodo**.
- The authors correctly emphasize the **high prevalence of emmetropia in IXT patients**.
- The need for **longitudinal studies** is acknowledged.

**Weaknesses & Required Revisions:**

- **Literature Citations:** Some **key references in strabismus research are missing**, and some citations are **misplaced or unsupported**.
- **Overinterpretation of Causality:**
  - The study **suggests that IXT may contribute to myopia development**, but this is not supported by longitudinal data.
  - *Suggestion:* The authors should **reframe their claims** to reflect an **association rather than causation**.
- **Data Interpretation Issues:**
  - The **symmetry of refractive errors between both eyes** is mentioned but **not statistically tested**.
- **Lack of a Control Group:**
  - The study **does not compare** refractive errors in IXT patients to children with normal binocular vision.
  - *Suggestion:* While retrospective studies cannot add new data, the authors should **compare their findings to published population-based studies**.
- **Selection Bias:**
  - The sample consists only of **clinic patients**, which may not be representative of the general population.
  - *Suggestion:* Acknowledge this limitation in the **discussion section**.
- **Lack of Extended Data & Data Dictionary:**
  - It is unclear whether the **shared dataset contains patient-level raw data or only summary statistics**.
- **Ethical Consent Issues:**
  - The study claims to be **retrospective**, yet **verbal consent was obtained**, which is unusual for retrospective research.
  - *Suggestion:* Clearly explain **how and why consent was sought**, especially for minors.
- **Lack of Statistical Comparisons:**
  - The study does **not use statistical tests to compare subgroups** (e.g., gender, age differences).
  - *Suggestion:* Perform **t-tests (or Mann-Whitney U) for continuous data** and **chi-square tests for categorical comparisons**.
- **No Multivariable Analysis:**
  - Potential confounders ( **screen time, family history of myopia**) are not accounted for.
  - *Suggestion:* Consider **logistic regression** to adjust for these factors.
- **Data Format is Unclear:**
  - It is unknown whether individual patient data is provided.
  - *Suggestion:* **Clarify dataset format** in the methods section.
- **Causal Claims Are Overstated:**
  - The study implies that IXT may **cause** myopia progression.
  - *Suggestion:* Revise statements to clarify that the findings suggest **an association, not causation**.
- **Lack of Context for Findings:**
  - Without a control group, the clinical significance of the **high emmetropia prevalence** is unclear.
  - *Suggestion:* Compare findings to **population-based refractive error studies**

Is the work clearly and accurately presented and does it cite the current literature?

Yes

If applicable, is the statistical analysis and its interpretation appropriate?

Partly

Are all the source data underlying the results available to ensure full reproducibility?

Yes

Is the study design appropriate and is the work technically sound?

Partly

Are the conclusions drawn adequately supported by the results?

Partly

Are sufficient details of methods and analysis provided to allow replication by others?

Partly

Reviewer Expertise:

Dispensing Optics, Contact Lens, Orthoptics, Low Vision, Anterior Segment, Posterior Segment, Refractive Error

I confirm that I have read this submission and believe that I have an appropriate level of expertise to confirm that it is of an acceptable scientific standard.

# Response

**Published:** 2025-03-31

**Authors:** Najah Mohammad

**Affiliations:** Department of Surgery, University of Baghdad, Baghdad, Baghdad Governorate, Iraq

Dear Reviewer,

 We sincerely thank you for the constructive comments. We have carefully addressed each point raised and revised the manuscript accordingly to enhance its clarity, rigor, and overall quality.

**Weaknesses and Required Revisions**

**Comment: Some key references in strabismus research are missing, and some citations are misplaced or unsupported.**

**Response:** Thank you for noting this. We have thoroughly reviewed and revised the reference list. We added several relevant and recent studies related to intermittent exotropia (IXT) and refractive errors in children, and corrected citations that were misplaced or lacked appropriate context (Line 201-207).

**Comment: The study suggests that IXT may contribute to myopia development, but this is not supported by longitudinal data.**

**Response:** We have revised all statements implying causality to instead reflect a possible association. In both the abstract and discussion, we now emphasize that our cross-sectional data can suggest correlation but not causation.

**Comment: Symmetry of refractive errors between both eyes is mentioned but not statistically tested.**

**Response:** While we did not conduct formal statistical testing, we clarified this in the Results and Discussion sections and rephrased the text to describe the observed pattern of bilateral consistency rather than making statistical claims.

**Comment: The study does not compare refractive errors in IXT patients to children with normal binocular vision.**

**Response:** We acknowledge this limitation. Since this is a retrospective clinic-based study, we did not have access to a matched control group.

**Comment: The sample consists only of clinic patients, which may not be representative of the general population.**

**Response:** A statement acknowledging this limitation has been added to the Discussion section, noting that findings should be interpreted cautiously and may not be generalizable to all pediatric populations.

**Comment: It is unclear whether the shared dataset contains patient-level raw data or only summary statistics.**

**Response:** We have clarified in the Methods section that the dataset shared on Zenodo includes de-identified patient-level data, including age, gender, right and left eye refractive status, and clinical classification of IXT.

**Comment: The study is retrospective, yet verbal consent was obtained, which is unusual for retrospective research.**

**Response:** Verbal assent was sought during follow-up visits for anonymized data use when feasible, in addition to retrospective chart review approved by the institutional ethics committee.

**Comment: The study does not use statistical tests to compare subgroups (e.g., gender, age differences).**

**Response:**  Regarding the suggestion to analyze the relationship between age, gender, and refractive status in our study. In response to this valuable feedback, we have now included age and gender subgroup analyses in the revised version.

**Comment: Confounders (screen time, family history) were not controlled for.**

**Response:** We appreciate the reviewer’s insightful comment regarding the role of potential confounders in the association between refractive error and intermittent exotropia (IXT). We acknowledge that several factors, including accommodative-convergence mechanisms, screen time, outdoor activity, family history, and near work, could influence both refractive status and IXT progression. However, our study was designed as an observational analysis focusing on refractive distributions in IXT patients, rather than a causal investigation of the factors contributing to IXT development.

**Comment: It is unknown whether individual patient data is provided.**

**Response:** We have revised the Methods and Data Availability Statement to clarify that individual, de-identified patient-level data are included in the Zenodo repository.

**Comment: The study implies IXT may cause myopia.**

**Response:** This point has been addressed above in Comment #2. All language suggesting causation has been revised to reflect association only.

**Comment: Without a control group, the clinical significance of the emmetropia prevalence is unclear.**

**Response:** We have added context in the Discussion by comparing our findings with published data from population-based refractive error studies in children from similar age groups and regions.

 Best Regard

 Najah

# Reviewer report: Reviewer response for version 1

**DOI:** 10.5256/f1000research.172316.r343840

**Published:** 2024-12-10

**Authors:** Jiawei Zhou, Yiqiu Lu

**Affiliations:** 1 School of Ophthalmology and Optometry, Eye Hospital, Wenzhou Medical University, Wenzhou, China

The author conducted a two-year cross-sectional study in an outpatient clinic of Najah Al-Quraishi and demonstrated that the most common refractive error among the children recruited was emmetropia. This study contains some interesting findings and are valuable for the understanding of refractive status in Iraqi children with intermittent exotropia. Overall, the article still needs some revisions in the description of its content and presentation of data, and I believe that the overall causal logic and clinical significance of the article is debatable.

1. The purpose of the study is not clearly stated in the abstract, and it is recommended to articulate it explicitly in the background section.
2. Incidence and prevalence are distinct statistical concepts, and it is inconsistent to use prevalence in the abstract’s results section and then switch to incidence in the conclusion. This study merely reports the proportion of existing refractive errors among participants, making it inappropriate to claim a high incidence of emmetropia. Prevalence would be the more accurate term. Furthermore, the classification of emmetropia as an illness is questionable.
3. The quality of the references is average and there seems to be no classic literature in the field of strabismus.
4. The authors mention in the introduction that many children with intermittent exotropia become myopic over time, but the literature cited is not a longitudinal study to draw this causal relationship.
5. The study’s purpose, based on its findings, should focus on the distribution of refractive errors in Iraqi pediatric and adolescent patients, rather than the treatment and prognosis of intermittent exotropia. Additionally, the last paragraph of the background does not flow logically.
6. Please clearly state the gaps or limitations of previous research, as I couldn’t identify them from the text.
7. In the first paragraph of “Statistical Analysis,” the phrase “the mean for both the spherical equivalence and the cylindrical and spherical correction” should use the precise term “spherical equivalent refraction (SER)” if that is what it refers to. If so, the refraction should have two decimal places and units.
8. The content under “Ethics and Consent” is duplicated in “Data Collection.”
9. In the Data collection section, orthopia should not be included in the grouping of low-moderate and high myopia, with low myopia being [-0.75D to -3.00), moderate myopia being [-3.00 to -6.00), and high myopia being -6.00 and above
10. The images in Figure 1 are duplicated in Figure 2; there’s no need to include both.
11. Fig1 should be a histogram distribution instead of heatmap, suggest to change it.
12. The use of punctuation in the key takeaway in Figure 2 should be more uniform and standardized.
13. It is suggested to revise Table 2 by adding “Refractive Error OD” and criteria for classifying refractive errors for improved clarity.
14. Please clarify whether the unit for refractive errors is eye or participant, as there is some confusion in the results section of the paper.
15. The results presented in “Symmetry in Refractive Errors” are not entirely convincing. They come across as too vague and generic, and there is a discrepancy between Figure 2 and this section of the results. Could you please do further analysis to conclude that the correlation is high?
16. The results of the detailed refractive error analysis should be presented in tables or plots for improved clarity.
17. The discussion section contains several instances of misplaced or missing citations. For example, the reference in the final sentence of the second paragraph does not correspond to the Pakistani study mentioned in the text. Revision is recommended.
18. In the fourth paragraph of the discussion section, the author states, “The paper focuses on the trend of an increase in myopia among children diagnosed with intermittent exotropia.” However, the evidence provided is neither sufficient nor convincing. Furthermore, the following conclusion that “This can indicate that developing myopia is not the cause of exotropia” cannot be supported based on this statement. It is recommended to adopt a less definitive tone.
19. In the fifth paragraph of the discussion section, the author states, “Given our findings, therapeutic strategies for addressing refractive errors in intermittent exotropia patients have to be tailored accordingly.” While this is an interesting premise, there is insufficient discussion on how or why such tailoring should be implemented.
20. In the final paragraph of the discussion, the conclusion that there is “a causative relationship between strabismus and myopia development” does not seem supported by the study’s results, and this conclusion cannot be considered to have “significant implications.” It is recommended to soften the tone. Additionally, the later mention of “missing longitudinal data defining the temporal development of refractive errors relative to the onset and progression of intermittent exotropia” contradicts this conclusion.

Is the work clearly and accurately presented and does it cite the current literature?

Partly

If applicable, is the statistical analysis and its interpretation appropriate?

Partly

Are all the source data underlying the results available to ensure full reproducibility?

Yes

Is the study design appropriate and is the work technically sound?

Partly

Are the conclusions drawn adequately supported by the results?

Partly

Are sufficient details of methods and analysis provided to allow replication by others?

Partly

Reviewer Expertise:

Amblyopia, Binocular vision, Visual plasticity

We confirm that we have read this submission and believe that we have an appropriate level of expertise to state that we do not consider it to be of an acceptable scientific standard, for reasons outlined above.

# Response

**Published:** 2024-12-15

**Authors:** Najah Mohammad

**Affiliations:** Department of Surgery, University of Baghdad, Baghdad, Baghdad Governorate, Iraq

We appreciate the time and effort you have taken to review our manuscript and for providing insightful comments and suggestions. We have carefully considered each point raised and have revised the manuscript accordingly. Below is a detailed response to your comments:

  

1. We have revised the abstract and background sections to explicitly state the purpose of the study. The purpose now highlights our aim to investigate the distribution of refractive errors in children with intermittent exotropia and its clinical implications.
2. We have replaced "incidence" with "prevalence" throughout the manuscript, as this study reports proportions rather than the emergence of new cases. Additionally, we clarified that emmetropia is not classified as an illness.
3. We carefully selected the cited literature to ensure it aligns with the focus and scope of the manuscript. While I aimed to include relevant and high-quality studies, I acknowledge that perspectives on what constitutes classic literature may vary. I did my best to include the most pertinent and available sources within the context of this work, and I hope the selection adequately supports the study's objectives. 
4. The introduction and discussion sections have been revised to adopt a less definitive tone. We now emphasize the need for longitudinal studies to establish causal relationships.
5. We have restructured the background to clarify the study's primary focus on refractive error distribution and removed extraneous emphasis on treatment and prognosis.
6. We have expanded the introduction and discussion sections to explicitly outline the gaps in prior studies, such as the lack of comprehensive data on refractive error profiles specific to intermittent exotropia and the absence of longitudinal analyses.
7. We have replaced "spherical equivalence" with "spherical equivalent refraction (SER)" where appropriate and included refractive measurements to two decimal places with units.
8. The overlapping content has been streamlined to remove duplication and improve clarity.
9. Orthopia has been excluded from the myopia classification, and we clarified the myopia ranges in the data collection section.
10. We have removed redundancy between Figures 1 and 2.
11. The data is categorical in nature, histogram is not suitable for such issues
12. Figure 2 has been removed
13. Table 2 has been revised to include a column for "Refractive Error OD" and a footnote detailing the refractive error classification criteria.
14. We have clarified in the methods and results sections that refractive errors are reported per patient, not per eye, to reflect the nature of the data.
15. Figure 1 has been replaced by a confusion matrix to better visualize the data
16. We have added a table to present the detailed refractive error analysis for better clarity.
17. We have corrected all misplaced citations and ensured they align with the referenced studies.
18. We have revised the statement to reflect the observational nature of the findings and removed unsupported causal implications.
19. We have expanded the discussion to include specific examples of tailored therapeutic strategies based on the study's findings.
20. The final paragraph has been revised to remove unsupported causal claims and emphasize the need for longitudinal studies.

We believe these revisions have strengthened the manuscript and addressed your concerns thoroughly. Thank you again for your valuable feedback.

 Sincerely,

# Reviewer report: Reviewer response for version 1

**DOI:** 10.5256/f1000research.172316.r339230

**Published:** 2024-11-28

**Authors:** Martin Ming-Leung Ma

**Affiliations:** 1 a State Key Laboratory of Ophthalmology, Zhongshan Ophthalmic Center, Sun Yat-sen University, Guangzhou, China

English editing is needed for this paper. Citations are lacking in some places. And there are some problems in how the authors analyse the data. The lack of written consent is concerning. I also did not understand how and when the verbal consent is obtained if it is a retrospective study. 

 Abstract:

 Background:

 “mostly in children aged 2-4 year”: clarification needed

 refractive mistakes: should be “refractive error”

 Results:

 “The most expected refractive error”?

 Manuscript:

 Introduction:

 “It is insidious since this outward deviation of an eye happens only once in a while, hence disturbing binocular vision “not true for all patients with IXT

 “The complicated relationship between intermittent exotropia and refractive errors has been known, though not so much understood; according to some studies, different patterns of refractive anomalies could exist in these patients.” Citation needed

 Method:

 Data collection:

 what is the dioptric definition of emmetropia?

 Author should stated it is a retrospective study in the abstract

 I don’t understand why there is a verbal consent if it is a retrospective study. I cannot comprehend what is meant by the “sensitive nature of the research topic”. If obtaining written consent from the participants is not feasible, why didn't the authors obtain it from their legal guardian or parents?

 Is there any tests for normality?

 Discussion:

 The main problem here is that the high prevalence of emmetropia in patients with intermittent exotropia should be viewed in context. 

 Author should also collect data regarding the distribution of refractive error types in patients with normal binocular vision. By comparing the distribution of refractive error in patients with and without IXT, we can then establish whether there is a relationship between these two factors. For instance, authors can take a look at this paper for reference: Ma, Martin Ming-Leung, et al. Ref 1.

Is the work clearly and accurately presented and does it cite the current literature?

Partly

If applicable, is the statistical analysis and its interpretation appropriate?

Partly

Are all the source data underlying the results available to ensure full reproducibility?

Yes

Is the study design appropriate and is the work technically sound?

Partly

Are the conclusions drawn adequately supported by the results?

Partly

Are sufficient details of methods and analysis provided to allow replication by others?

Yes

Reviewer Expertise:

Binocular vision

I confirm that I have read this submission and believe that I have an appropriate level of expertise to state that I do not consider it to be of an acceptable scientific standard, for reasons outlined above.

## References

[1] : Vergence and Accommodative Dysfunctions in Emmetropic and Myopic Chinese Young Adults. *J Ophthalmol* .2019;2019: 10.1155/2019/5904903 5904903 10.1155/2019/5904903 31396411 PMC6664731

# Response

**Published:** 2024-12-01

**Authors:** Najah Mohammad

**Affiliations:** Department of Surgery, University of Baghdad, Baghdad, Baghdad Governorate, Iraq

Thank you for your valuable feedback. We hope that the following changes address your concerns.

 We have revised the abstract as follows:

- Clarified the age group to "predominantly in children aged 2 to 4 years."
- Replaced "refractive mistakes" with "refractive errors."
- Rephrased "The most expected refractive error" to "Emmetropia was the most commonly observed refractive status."

We have addressed your concerns in the introduction section as follows:

- Revised the statement “It is insidious since this outward deviation of an eye happens only once in a while, hence disturbing binocular vision” to reflect that this is not true for all patients with intermittent exotropia (IXT).
- Added appropriate citations for the statement regarding the relationship between intermittent exotropia and refractive errors.

We have addressed the concerns regarding the methodology as follows:

- Added the dioptric definition of emmetropia (±0.50 diopters).
- Updated the abstract and methods to explicitly state the study's retrospective nature.
- Clarified the consent process, specifying verbal consent for additional data collection and parental/guardian consent for minors.
- Included normality testing using the Shapiro-Wilk test in the statistical analysis section.

We have made the following adjustments in the discussion section:

- Discussed the high prevalence of emmetropia in the context of refractive error distribution in normal binocular vision.
- We acknowledged the retrospective nature of our study, which limits the inclusion of a control group. We have suggested that future prospective studies include such comparisons to further explore the relationship between refractive errors and intermittent exotropia.
